# Factors associated with obesity alter matrix remodeling in breast cancer tissues

**DOI:** 10.1117/1.JBO.25.1.014513

**Published:** 2020-01-25

**Authors:** Yang Zhang, Fatma Kucuk Baloglu, Lauren E. Hillers Ziemer, Zhiyi Liu, Boyang Lyu, Lisa M. Arendt, Irene Georgakoudi

**Affiliations:** a Tufts University, Department of Biomedical Engineering, Medford, Massachusetts, United States; b Giresun University, Department of Biology, Giresun, Turkey; c University of Wisconsin–Madison, Department of Comparative Biosciences, Madison, Wisconsin, United States; d Zhejiang University, State Key Laboratory of Modern Optical Instrumentation, College of Optical Science and Engineering, Hangzhou, Zhejiang, China; e Tufts University, Department of Electrical Engineering, Medford, Massachusetts, United States; f Tufts University, Program in Cell, Molecular & Developmental Biology, Graduate School of Biomedical Sciences, Boston, Massachusetts, United States

**Keywords:** breast cancer, obesity, second-harmonic generation, two-photon excitation fluorescence, collagen fiber organization, three-dimensional variance, gray-level co-occurrence matrix

## Abstract

Obesity is associated with a higher risk of developing breast cancer and with worse disease outcomes for women of all ages. The composition, density, and organization of the breast tissue stroma are also known to play an important role in the development and progression of the disease. However, the connections between obesity and stromal remodeling are not well understood. We sought to characterize detailed organization features of the collagen matrix within healthy and cancerous breast tissues acquired from mice exposed to either a normal or high fat (obesity inducing) diet. We performed second-harmonic generation and spectral two-photon excited fluorescence imaging, and we extracted the level of collagen-associated fluorescence (CAF) along with metrics of collagen content, three-dimensional, and two-dimensional organization. There were significant differences in the CAF intensity and overall collagen organization between normal and tumor tissues; however, obesity-enhanced changes in these metrics, especially when three-dimensional organization metrics were considered. Thus, our studies indicate that obesity impacts significantly collagen organization and structure and the related pathways of communication may be important future therapeutic targets.

## 1 Introduction

Obesity is a worldwide pandemic, and it is considered as high risk for numerous diseases.^1^ For women with breast cancer, obesity is typically associated with both a worse initial diagnosis and shorter disease-free survival rates than nonobese patients.^2^ Recent studies are beginning to reveal interesting connections between obesity, hypoxia, inflammation, and the characteristics of the adipose tissue stroma within healthy and cancerous breast tissues.^3^ Parameters that affect stromal micromechanical properties, such as collagen content, organization, and cross-links, are of particular interest since important mechanosignaling pathways have already been identified that affect disease development and metastasis.^4^^–^^8^ Further, tissue stiffening and increases in mammographic tissue density are established breast cancer diagnostic markers. The content and organization of collagen fibers are key contributors to tissue stiffness and density and distinct tumor-associated collagen organization features have been identified at various stages of breast cancer development.^9^ Obesity has been shown to affect collagen organization, while diabetes and obesity are also broadly correlated with enhanced tissue stiffening that is likely mediated by an enhanced level of advanced glycation end-product (AGE) cross-links. However, the impact of obesity on collagen fiber organization and collagen-associated fluorescence (CAF), especially, in the context of breast cancer have not been examined yet.

Second-harmonic generation (SHG) imaging is ideally suited to provide details on the three-dimensional (3-D) organization of collagen fibers in thick tissue specimens in a nondestructive, label-free manner, which relies on the unique noncentrosymmetric structure of collagen fibers as a specific source of optical imaging contrast.^10^ This method has been used extensively to characterize collagen fibers in the context of numerous diseases, including breast cancer.^11^^–^^14^ Our group has developed a fast, automated, and accurate approach to assess 3-D fiber orientation based on weighted vector summation, and we have established the 3-D variance as a sensitive, quantitative metric of fiber organization.^15^^,^^16^ The 3-D variance is calculated using the polar and azimuthal angles that define fiber orientation within an image stack, and it varies between 0 (for perfectly aligned fibers) and 1 (for completely randomly oriented fibers).^16^ In previous studies, we have shown that the 3-D variance of collagen fibers is affected by the presence of hormones during mammogenesis and decreases within breast tumors when compared to healthy mammary tissue.^16^^,^^17^ We have further demonstrated that the 3-D variance is a more sensitive indicator of collagen fiber organization changes than corresponding metrics extracted from the analysis of stacks of two-dimensional (2-D) images.^18^

Collagen cross-links have also been shown to affect tissue micromechanical stiffness.^6^^,^^19^^–^^22^ Enzymatic cross-links are mediated by lysyl hydroxylase and lysyl oxidase (LOX), whereas nonenzymatic cross-links are typically referred to as AGEs and are formed through glycosylation or glycation.^23^^–^^25^ Increased LOX-mediated collagen cross-links lead to elevated tissue fibrosis and stiffness in the context of both obesity and breast cancer,^3^^,^^26^^,^^27^ whereas upregulated AGE-mediated collagen cross-links have been found to stiffen the ECM in patients and animal models of diabetes mellitus and aging.^28^ Both of these types of cross-links are associated with fluorescent emission. While LOX-induced pyridinoline cross-links are typically associated with fluorescence emission that has a maximum at 400 nm, AGE-related cross-links with emission maxima at 385, 405, 440, and 510/520  nm have been reported.^29^^–^^33^

In this study, we sought to examine the effects of high-fat diet (HFD)-induced obesity on the collagen fiber organization and fluorescence of mammary tissues in both tumor-bearing and nontumor-bearing mice. We acquired 3-D SHG image stacks at 920-nm excitation/460-nm emission and 2-D two-photon excited fluorescence (TPEF) spectral images at 740- and 860-nm excitation and 390- to 750-nm emission. We analyzed these images using automated algorithms to extract quantitative metrics of 3-D fiber alignment and content, 2-D collagen texture features, and CAF. We report on the presence of significant differences in several of these metrics between normal and tumor tissues; notably, differences in 3-D alignment and CAF are enhanced in the context of obesity.

## 2 Materials and Methods

### 2.1 Tissue Collection

Animal procedures were performed in accordance with a protocol approved by the University of Wisconsin–Madison Institutional Animal Care and Use Committee. Mice were housed and handled in AAALAC-accredited facilities in accordance with guidelines from the National Institutes of Health (NIH) Guide for Care and Use of Laboratory Animals. All female mice were purchased from Jackson Laboratories (C57Bl/6J, 00064). In this study, twenty 8-week-old female C57Bl/6 mice were fed either a control (normal) diet (ND, 5% kcal from fat; Teklad Global #2018) (n=10) or HFD (60% kcal from fat, Test Diet #58126) for 16 weeks to induce obesity (n=10). Purified diets contained equal amounts of vitamins and micronutrients. All mice were given water and the same chow *ad libitum*. To generate tumors, $1\times 10^{6}$ EO771 cells, which were derived from a spontaneous adenocarcinoma in a C57Bl/6 mouse,^34^ were suspended in matrigel and injected into the inguinal mammary glands of HFD (n=5, HT) or ND-fed (n=5, NT) mice.

The healthy tissue samples comprised the entire fourth mammary gland and were $∼500\;{\mathrm{mm}}^{3}$ in size. When tumors reached 1.5 cm in diameter, mice were euthanized and a quarter of the tumor, $∼130\;{\mathrm{mm}}^{3}$ in size, including both the edge as well as the interior of the tumor, was sectioned. Samples were snap frozen immediately upon excision in optimal cutting temperature (OCT) medium in a bath of methanol and dry ice. Once frozen, the blocks were stored at −80°C and sent on dry ice to Tufts, where they continued to be stored in a −80°C freezer until imaging. An identical protocol was followed for tissue freezing and all samples were shipped to Tufts at the same time. All samples were imaged within a period of a month from each other.

### 2.2 Imaging

An identical protocol was followed for tissue thawing and preparation prior to imaging. Specifically, each tissue was cut in half immediately after being taken out of the −80°C freezer, and half of the sample together with OCT were placed in a petri dish, allowed to thaw for at least 5 min at room temperature, then washed in phosphate-buffered saline (PBS) twice to remove the OCT. The other half was placed back in the −80°C freezer. The thawed tissue was placed on a glass-bottom dish with the incision side placed against the coverslip for imaging. To keep the samples hydrated, one drop of PBS was added on top of each sample.

Images (512×512  pixels; 387.5×387.5  μm) of the excised breast tissues were obtained using a Leica TCS SP8 confocal microscope equipped with a tunable (680 to 1300 nm) femtosecond laser (InSight Deep See; Spectra-Physics; Mountain View, California) and a water-immersion 40× objective (NA 1.1). The collagen SHG signals were measured using 920-nm excitation and collected by an external HyD detector with a 460±20-nm emission band-pass filter. A 1162.5-μm×1162.5-μm composite image stack, which is composed of nine adjacent stacks, was acquired from each sample over a depth of 80  μm with a step of 2  μm. Spectral images were collected using 740- and 860-nm excitation wavelengths, over emission wavelengths in the 390- to 750-nm range, with a detection bandwidth of 10 nm, using an internal HyD detector. Spectra from 12 fields were collected for each of the NN and HN groups, while 18 spectral images were acquired from each of the NT and HTgroups. Spectra and spectral images were acquired from five independent tissue blocks from each of the HN, NT, and HT groups, and four independent tissue blocks from the NN group. One of the NN tissue blocks was used to optimize image acquisition parameters. To minimize bias introduced in our calculations based on the specific regions that we imaged, we selected our imaging regions following a 2-D tile scan that covered at least a 5-mm×5-mm region of the tissue and selected an area that represented the main tissue features (e.g., fiber rich versus fatty rich regions).

### 2.3 Image Processing and Analysis

#### 2.3.1 3-D variance analysis

All acquired images were normalized for detector gain and day-to-day variations in illumination power. Collagen fiber organization was calculated based on 3-D stacks of SHG images using an algorithm we described in detail previously.^15^ Briefly, the approach relies on weighted vector summation over a 3-D window whose size may be optimized for a particular set of samples to identify a dominant fiber direction, followed by calculations that define the polar and azimuthal angle of the fiber and the variance in the corresponding orientations. SHG-positive image pixels were determined using the 920/460-channel where SHG signal dominated; image background was removed using 2% of the maximum image intensity threshold and Otsu’s thresholding method.^35^ Five different localized window sizes ranging from $3.8\times 3.8\times 6\;\mu m^{3}$ to $387.5\times 387.5\times 80\;\mu m^{3}$ were used to assess organizational differences at different scales. To better characterize the 3-D variance of the collagen fiber alignment, we use the kernel density estimation (KDE) method to plot the distribution of 3-D variance values of all voxels within a stack in terms of a probability density function (PDF).^36^ For each stack, we calculated the mean 3-D variance, the peak, skewness, and full-width at half-maximum (FWHM) of the 3-D variance distribution. We used the Kullback–Leibler divergence (KLD) algorithm to measure the dissimilarity between any two PDFs and compared the mean KLD values for each group pair. When two PDFs are identical, the KLD value is zero while increasing values correspond to increasing levels of difference between two distributions.

#### 2.3.2 Two-dimensional collagen texture analysis

As additional metrics of 2-D collagen organization, we assessed the texture of the SHG images using the gray-level co-occurrence matrix algorithm (GLCM).^37^ This statistical approach considers the spatial relationships of intensity fluctuations within two pixels of an image at a time, called the reference and the neighbor pixels. In this study, for each image pixel, we considered relationships with 12 increasing pixel distances (1, 2, 4, 6, 8, 10, 12, 14, 16, 18, 20, and 25 pixels) and four orientations (0 deg, 45 deg, 90 deg, and 135 deg). Information from the corresponding supplementary angles (e.g., 180 deg, 225 deg, 270 deg, and 315 deg) was incorporated simultaneously, since the GLCM was considered to be symmetric. To avoid zero intensity image gaps that can affect the statistical calculations, we digitally cloned the image intensities contained within SHG-positive areas (assessed as in Sec. 2.3.1) into any zero intensity image gaps, using a custom automated digital object cloning technique we described previously.^38^^,^^39^ This is done in a random manner to ensure that there is no user bias in preferentially selecting one region of the image to fill in the segmented gaps. The SHG-positive pixels are not overwritten to preserve the inherent textural heterogeneity of an image. We computed the correlation to evaluate the spatial distribution of intensity variations within each cloned image. We reported the $D_{50}$ value, the distance at which the mean correlation value declines to 50% of its initial value, as a metric of the level of intensity correlations, which depends on the organization and the relative size of collagen fibers in each group. The $D_{50}$ values for all the 2-D images of a tissue sample were transformed to PDFs and compared using the KLD method.

#### 2.3.3 Spectroscopic data analysis

SHG-positive pixels were considered any pixels with nonzero intensity values following integration of images collected at 420, 430, and 440 nm emissions acquired at 860 nm excitation. The corresponding mask for each field was applied to all emission sequences acquired at 740- and 860-nm excitation wavelengths. To quantify the fluorescence signal emanating from the SHG-positive pixels, we integrated all the corresponding pixel intensities within each emission band to generate a spectrum from a given imaged field. The intensity of each spectrum was normalized by the intensity of the SHG signal peak value (detected at 860-nm excitation/430-nm emission) for both wavelengths. The alternating least square algorithm provided in the Matlab PLS toolbox (Mathworks Inc., Natick, Massachusetts) was used to deconvolve the spectra into the spectral shapes and corresponding weights of two components.

### 2.4 Tissue Staining

Hematoxylin and eosin (H&E) and anti-α-smooth muscle actin (SMA) stainings were performed on both tumor and nontumor tissues by UWCCC Experimental Pathology Laboratory. H&E staining was used to evaluate the tissue structures while α-SMA is a marker for cancer-associated fibroblasts, which could be one source of collagen remodeling within the tumor microenvironment. Tumors were fixed in 10% neutral buffered formalin for 24 to 48 h, embedded in paraffin and sectioned at 5  μm. Paraffin-embedded sections were rehydrated in graded alcohol and incubated with 0.5% $H_{2}O_{2}$ for 20 min. Sections underwent antigen retrieval in 0.01 M citrate buffer (pH=6.0) at 95°C for 20 min and stained using the M.O.M. Kit (Vector BMK-2202) according to package instructions. Tissue sections were incubated with anti-α-SMA antibodies (Sigma Aldrich, A2547) followed by Alexa Fluor 546 goat anti-rabbit IgG (Life Technologies, A11010), then counterstained with 250  ng/ml 4′,6-diamidine-2′-phenylindole dihydrochloride (DAPI) for 5 min. Slides were mounted with the Slow-Fade mounting kit (Life Technologies, S2828). Sections were imaged using a Nikon Eclipse 80 t microscope with NIS Elements BR 3.2 software.

### 2.5 Statistics

All image processing was performed in MATLAB_R2018a software. Statistical analysis was performed in JMP Pro 14 (SAS Institute, Cary, North Carolina). Significance in the difference of the values of the different metrics among different groups was calculated based on one-way ANOVA and post-hoc Tukey tests. Comparisons were performed to assess differences either as a result of the presence of tumor or in response to diet (i.e., NN was compared to HN and NT groups, HN was compared to NN and HT, NT was compared to NN and HT). Significant differences at α=0.05 or lower are reported. Five parameters (minimum, lower quantile, median, higher quantile, and maximum) were used to generate the box plots, and mean ± standard error of the mean were used for the bar graphs.

## 3 Results

### 3.1 Collagen Fibers Are More Aligned in Tumor Tissues than in Normal Tissues, while Obesity Further Enhances the Alignment Level Preferentially in Tumor Tissues

**Fig. 1:**
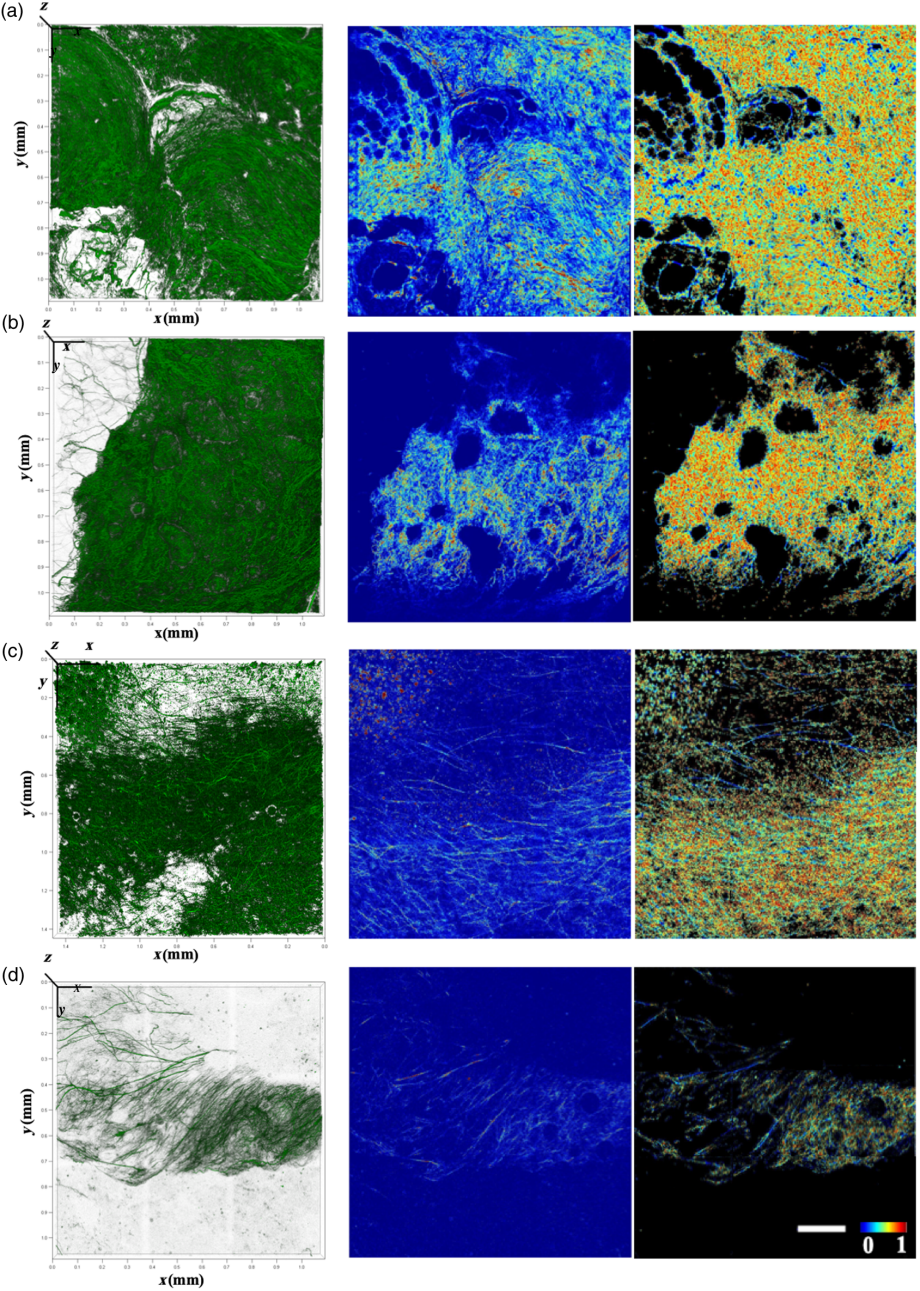
Representative volume renderings (pseudocolored green) (left column), 2-D optical sections (middle column) and corresponding 3-D variance color-coded collagen fibers (right column) from the four different tissue types. (a) NN, (b) HN, (c) NT, and (d) HT. Scale bar and color bar are the same for the panels in columns 2 and 3. Scale bar=200  μm.

Representative SHG stacks (1162.5  μm×1162.5  μm×80  μm) and SHG optical sections (1162.5  μm×1162.5  μm) from tumor- and nontumor-bearing breast tissues from mice fed either a normal or HFD are shown in Fig. 1. The presence of more highly aligned and less dense fibers in tumor bearing tissues is easily perceived from these images. The corresponding 3-D variance coded optical sections enhance visualization of these features, with fibers represented by bluer hues (lower variance/better alignment) and occupying a smaller fraction of the field in the tumor tissues. The dotted region in the upper left corner of the NT (normal diet fed, tumor) images corresponds to cross-sections of fibers oriented primarily along the transverse direction, as indicated more clearly in Fig. S1 in the Supplementary Material.

**Fig. 2:**
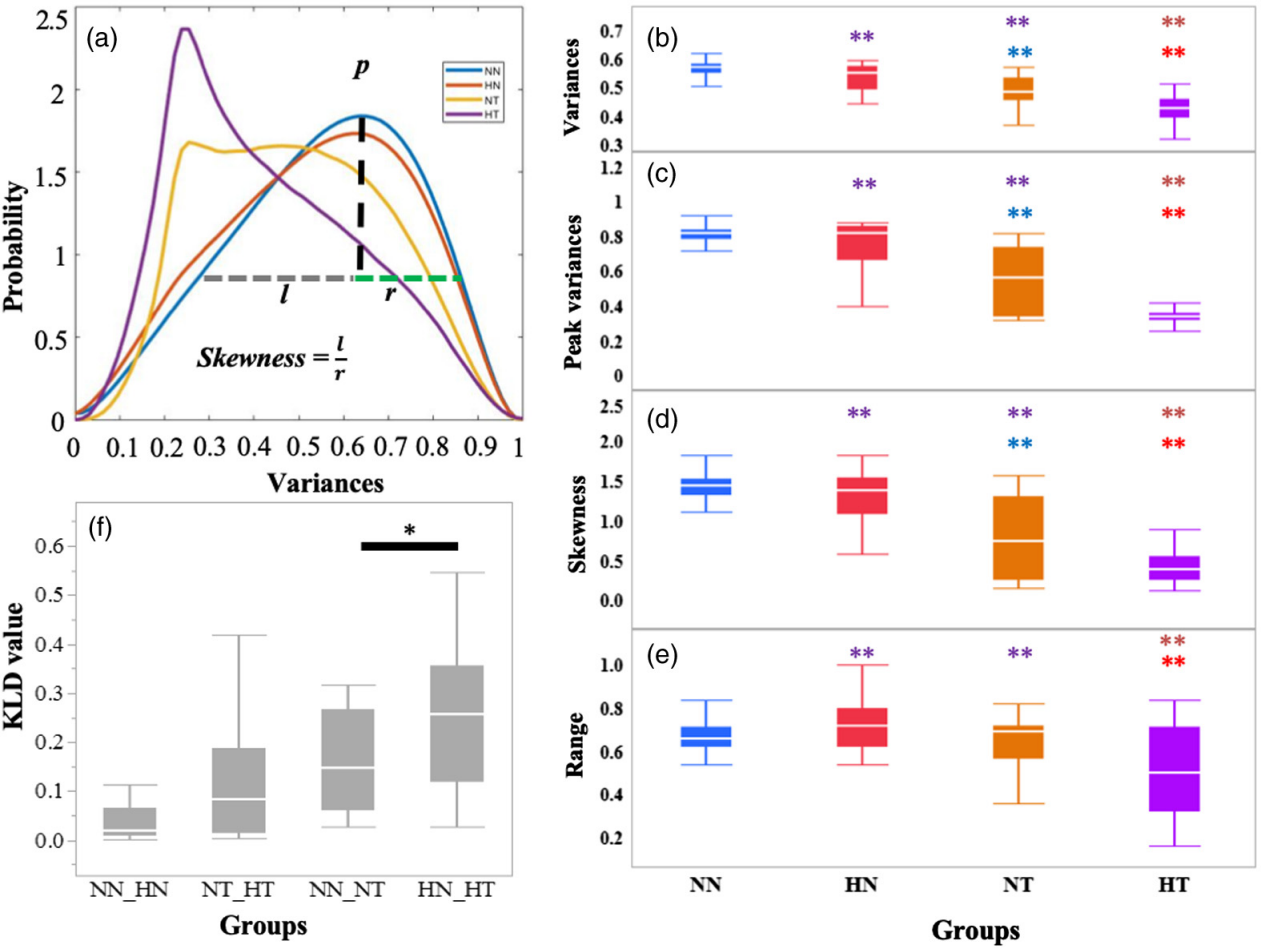
(a) Averaged PDFs of the voxel-wise 3-D collagen fiber variance after KDE. (b) The mean value, (c) the peak of the distribution, (d) its skewness, and (e) the FWHM range are shown. * and ** denote significance at a=0.05 and a=0.01, respectively. The color with which ** is indicated denotes the comparison group. (f) KLD values for corresponding group comparisons. Each box plot conveys the median, the first and third quantiles, and the minimum and maximum values.

The changes in alignment are quantified first using the 3-D stacks and the corresponding 3-D directional variance metrics (Fig. 2), calculated using a $5.3\times 5.3\times 6\text{-}\mu m^{3}$ localized window, which yielded significant levels of differences among different tissue types (Fig. S2 in the Supplementary Material). The decrease in sensitivity of the 3-D variance to report on differences between some of the groups as the window size increases reflects the presence of heterogeneities in alignment that are characterized more accurately with smaller window sizes. The averaged PDFs of the localized 3-D variance values of each sample within a group are shown in Fig. 2(a), clearly demonstrating significant shifts in the shape of these distributions toward smaller values within tumor tissues (NT and HT) compared to normal ones (NN and HN). While the distributions for normal breast tissues appear fairly similar independent of the diet to which the mice were exposed to (NN and HN), the shape of the 3-D variance PDF corresponding to tumor tissue from mice fed a HFD (HT) is even further weighted toward smaller values when compared to the NT one. The mean 3-D variance, the PDF’s peak 3-D variance value, its skewness (defined as the ratio of the widths to the right and the left of the location where a perpendicular drawn from the peak of the distribution crosses the FWHM line), and FWHM range report these trends [Figs. 2(b)–2(e)]. 3-D variance PDF shapes vary considerably among optical sections from a given tissue group and all the PDFs are shown in Fig. S3 in the Supplementary Material. Within the normal tissues, we observe that fields that include a duct or a significant number of adipocytes tend to have broader and/or low-value shifted PDFs. While it is harder to observe variations in the PDF shapes of the tumor tissues that depend on a somewhat consistent fashion on the level of cellularity or adipocity, it is clear that these PDFs are shifted to lower 3-D variance values when compared to the normal tissues, and we see such shifts more consistently for the HFD-fed tumor-bearing mice. The KLD values reported in Fig. 2(f) illustrate that the 3-D variance PDF differences are highest for the tumor versus normal comparisons (NN versus NT and HN versus HT) with the HFD leading to significant enhancement in those differences.

### 3.2 Tumor Tissues Have Lower Collagen Fiber Density and Less Correlated SHG Texture Features

**Fig. 3:**
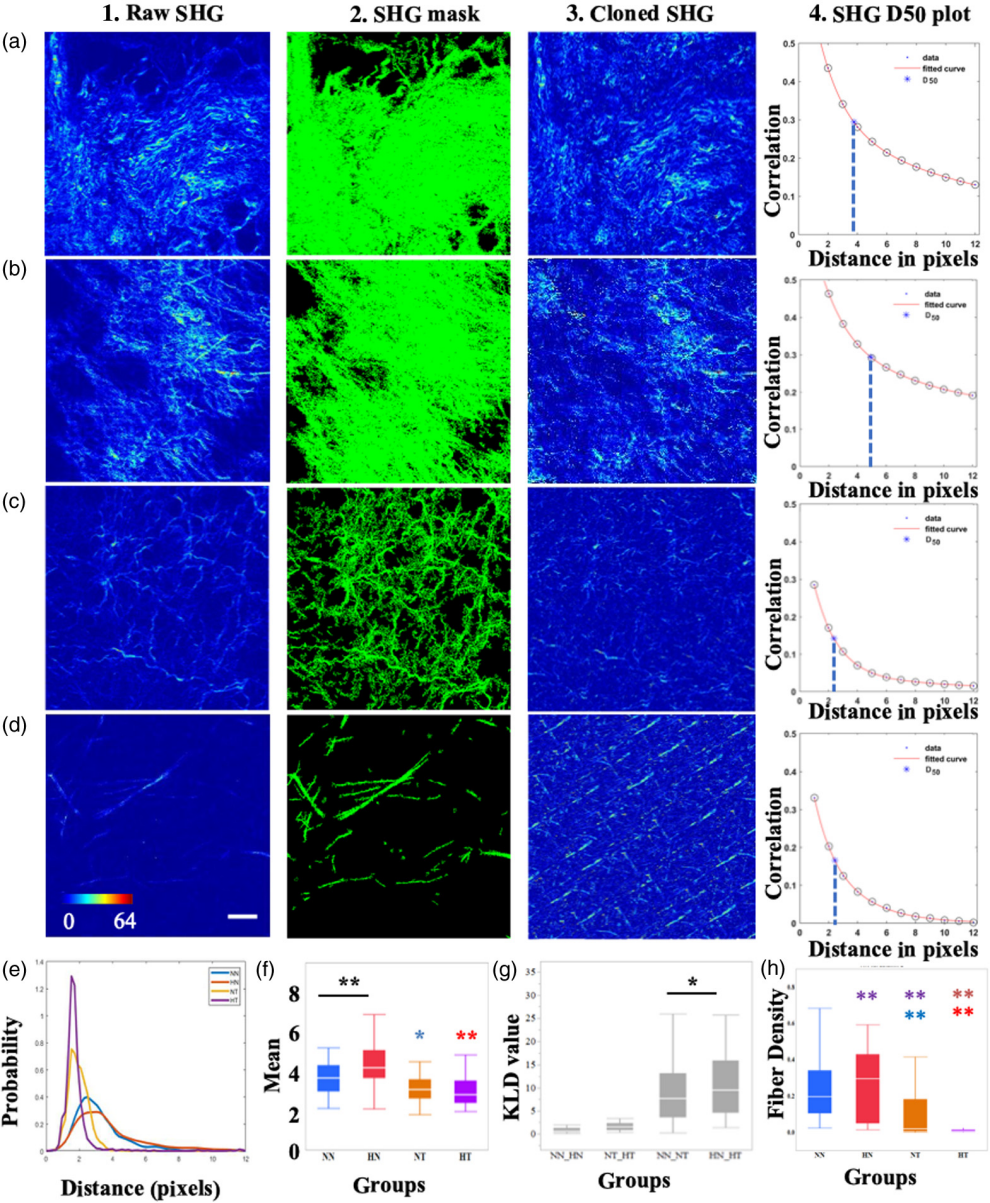
Textural analyses and representative figures. Representative detected SHG intensity images from each tissue group (a1-d1), along with corresponding SHG-positive masks (a2-d2), cloned SHG images (a3-d3), and correlation intensities as a function of pixel distance (a4-d4). (a1-a4) Normal diet, no tumor; (b1-b4) HFD, no tumor; (c1-c4) normal diet, tumor; (d1-d4) HFD, tumor. (1  pixel≈0.76  μm). Asterisks and blue lines signify for each plot the distance where correlation value declines to 50% of its initial value (D50) for the corresponding image. (e) PDF of D50 values from all optical sections assessed for each treatment group. (f) Box plots of the mean D50 values of each group. (g) KLD values of each D50 PDF group pairs. (h) Fiber density within the image stacks assessed for each group. Scale bar= 50  μm; colorbar and scalebar are the same for columns (1) to (3), panels (a)-(d). * and ** denote significance at a=0.05 and a=0.01, respectively. The color with which ** is indicated denotes the comparison group. The intensities of images listed in columns (1) and (3), panels (a)-(d) were rescaled and separated into 64 different intensity levels, which were assigned with different pseudocolors to range from blue to red.

While the HFD-fed mice in our studies gained significantly more weight than those fed the ND [Fig. S4(A) in the Supplementary Material], and the tumor weights in HFD-fed mice were significantly larger than those from ND-fed mice [Fig. S4(B) in the Supplementary Material], the tumors appeared similar for the different diet-treatment groups using H&E staining [Figs. S4(C)–S4(D) in the Supplementary Material], as well as the expression of positive cancer-associated fibroblasts via immunohistochemical α-SMA staining [Figs. S4(E) and S4(F) in the Supplementary Material]. Thus, these traditional 2-D staining procedures did not suggest differences in extracellular matrix remodeling. To further characterize 2-D SHG image texture, we used a GLCM-based approach. Representative SHG optical sections, along with corresponding cloned SHG images are shown in Fig. 3. From the SHG segmented images [Figs. 3(a2)–3(d2)], it is easy to perceive a significant decrease in fiber density in the tumor tissues of the breast cancer animal model we employed for this study. This decrease appears to be enhanced with obesity (i.e., HFD), as shown quantitatively in Fig. 3(h), where fiber density is reported as the ratio of SHG-positive pixels over the number of all the pixels within each SHG stack acquired from each tissue. A significant decrease in the $D_{50}$ value extracted from correlation analysis of the SHG image texture features is also highlighted in Figs. 3(a4)–3(d4), resulting probably from a combination of changes in the density and size of collagen fibers. Significant differences in the $D_{50}$ value PDFs for the different tissue types from all the images we acquired are shown in Fig. 3(e) and quantified by corresponding differences in the mean values of these distributions [Fig. 3(f)]. There are significant differences between tumor- and nontumor-bearing groups (NT versus NN and HT versus HN); however, an HFD does not lead to significant differences between the HT and NT groups, unlike the 3-D variance metrics [Fig. 3(g)]. Nevertheless, the KLD comparisons indicate that differences are indeed higher between the HN and HT groups than the NN and NT groups.

### 3.3 Collagen Associated Fluorescence is Significantly Higher within Breast Tumor Tissues of Obese Mice

**Fig. 4:**
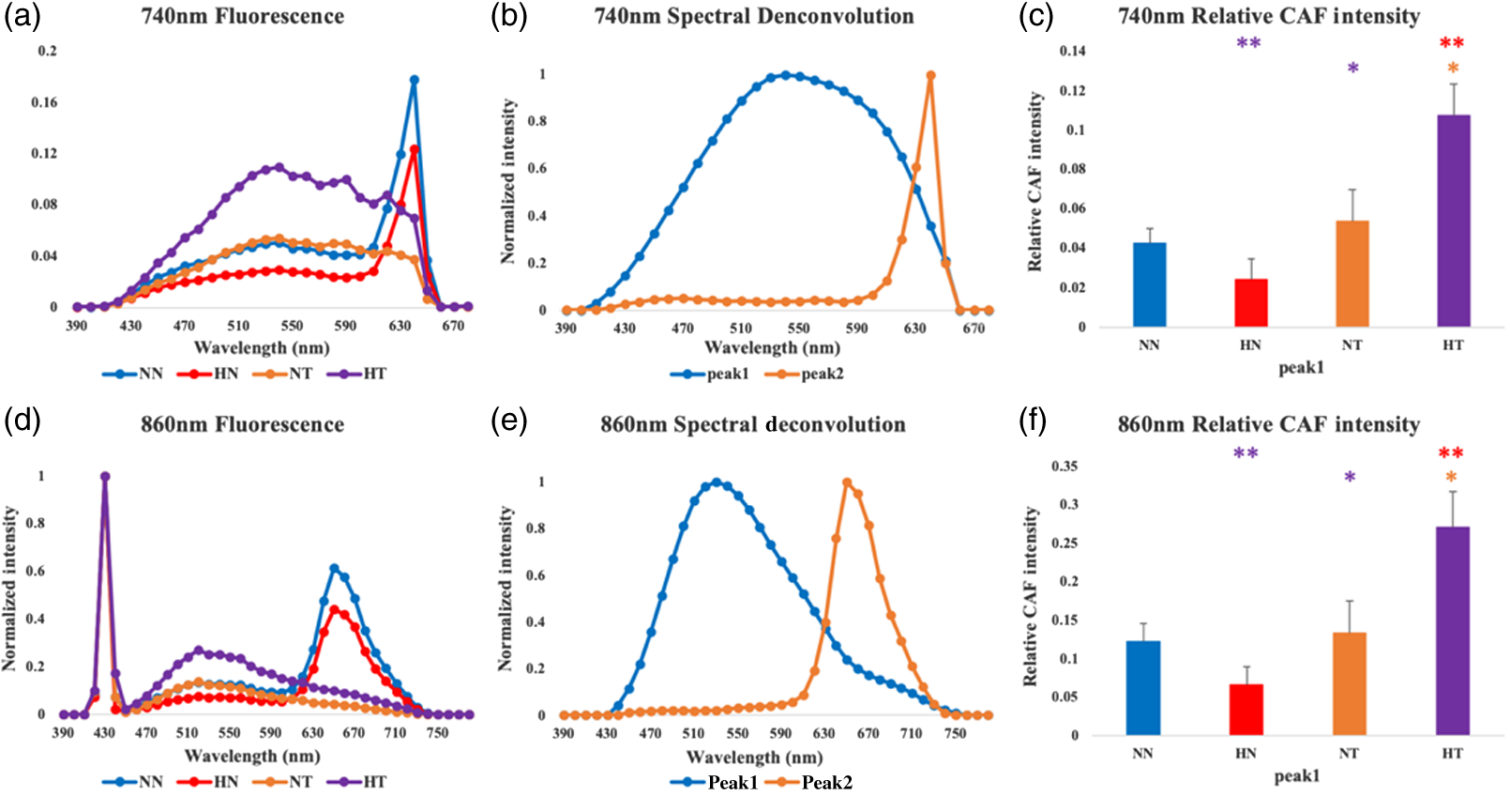
Graphs of CAF spectra at (a) 740 nm and (d) 860 nm excitation acquired with emission in the 390- to 790-nm region (steps: 10 nm) for NN, HN, NT, and HT groups. Spectral deconvolution results of the emission spectra for (b) 740 nm and (e) 860 nm excitation. Values for the weights of peak 1 in each group are shown in (c) and (f) as mean ± standard error of the mean (SEM). (*a=0.05, **a=0.01). The color with which * is indicated denotes the comparison group.

Average fluorescence emission spectra from each group of breast tissues we considered are shown from the SHG-positive pixels for 740- and 860-nm excitation in Fig. 4. The SHG peak is present at 430-nm emission within the 860-nm excitation spectra, as expected. All spectra were normalized to their corresponding 430-nm emission peaks so that the fluorescence signal intensity is effectively normalized to the collagen content, representing essentially a measure of fluorescent collagen-cross-link density. Especially, for nontumor-bearing mice, there is a strong peak in the 600 nm range of the spectrum, which likely emanates from porphyrins in the chow used to feed the mice and has been reported previously.^40^^,^^41^ While all mice were fed the same chow, the absence of strong porphyrin contributions within the tumor tissues is likely associated with changes in heme biosynthesis, which have been reported for a number of related enzymes for several cancers, including human breast cancers.^42^ To remove contributions from this peak, we performed spectral deconvolution to extract the shapes of the fluorescence emission that describe the spectra, which are shown in Figs. 4(b) and 4(e). The porphyrin spectral shape contributions are consistent for the two excitation wavelengths; however, the remaining fluorescence emission contributions have distinct shapes for the two excitation wavelengths, with the spectrum acquired at 740-nm excitation having broader features. This likely indicates the presence of more than one fluorophore in the tissue. However, the overall fluorescence intensity of this nonporphyrin-like emission follows very similar trends for the different tissue types for both wavelengths, as shown in Figs. 4(c) and 4(f). These panels indicate that obesity increases significantly the fluorescence that is present within tumor tissues selectively, and this increase leads to the detection of statistically different levels of fluorescence between tumor and nontumor tissues in the case of the HFD-fed mice, as well as between the tumor tissues from the mice fed with HFD or normal diet.

## 4 Conclusions and Discussion

Our goal was to assess the impacts of obesity on metrics of collagen organization and structure within the matrix of normal and tumor breast tissues, as assessed by quantitative analysis of high-resolution SHG and TPEF spectroscopic images. We identified metrics of 3-D orientation/alignment (3-D directional variance), 2-D organization ($D_{50}$ correlation distance), fiber density, and CAF intensity. We found that while multiple metrics of collagen characteristics were altered within tumor tissues when compared to normal breast, the presence of obesity impacted in more significant ways the stroma of tumor than that of nontumor-bearing mice. Metrics of 3-D collagen fiber organization reported more significant changes than those of 2-D organization and CAF. Nevertheless, the combination of features extracted from SHG and TPEF imaging may ultimately provide a more thorough picture of alterations in collagen architecture and could serve as a sensitive tool in important studies that aim to understand the role of obesity and collagen remodeling in breast cancer development and metastasis.

The increase in the levels of collagen fiber alignment (Fig. 2) and CAF (Fig. 4) that we observe within tumor tissues compared to normal breast tissues is consistent with a number of studies performed with animal models and human tissue specimens.^3^^,^^43^^,^^44^ Enhanced collagen fiber alignment, especially perpendicular to the tumor border, has been reported in several studies and is associated with enhanced metastatic potential, as the fibers are thought to serve as a conduit for tumor cells that are shed from the primary tumor and ultimately form metastases.^43^^,^^44^ Such changes in alignment, along with enhanced collagen fiber cross-links, are also associated with increased tissue stiffness, which has been shown to activate important mechanosignaling cellular pathways that promote malignancy, invasion, and metastasis.^6^^,^^45^^–^^48^

Statistically significant increases in fiber alignment and CAF are further enhanced within tumors excised from obese mice fed an HFD compared to tumors developed in mice fed a normal diet (Fig. 4). Our findings are in agreement with recent studies, which reported that collagen fibers assessed via analysis of 2-D SHG images acquired from human tissue specimens were more aligned within tumors of obese patients compared to those from patients with a normal body mass index.^3^ However, the same study also reported significant differences in the collagen organization of normal breast tissues derived from obese and nonobese patients, and our observations did not yield any such significant changes. This may be related to the method via which obesity was induced in the mice we studied.

In principle, obesity and diabetes have been associated with matrix stiffening that may be at least partially mediated by AGE cross-links.^49^^,^^50^ Emission from such cross-links is more consistent with the excitation/emission wavelengths of our study, than LOX-mediated cross-links. However, the impact of AGE cross-links on mechanical properties may be highly dependent on the type of tissue and the nature of the cross-links. While, in principle, one would expect that more cross-links would lead to stiffer tissues, it has been shown that AGEs can interfere with the formation of LOX-mediated cross-links^51^ and in the formation of collagen fibrils,^52^ which could in turn destabilize the matrix. In addition, very interesting studies are emerging that reveal important relationships between obesity, inflammation, and matrix remodeling, especially in the context of breast cancer.^53^ However, some of the interactions may lead to matrix degradation^54^^–^^58^ and others to enhanced cross-links.^6^ In fact, a recent study showed that levels of collagen fiber alignment, macrophages, and inflammatory markers are predictors of overall survival for breast cancer patients. Further studies could explore the more detailed relationships between two-photon-excited CAF, fiber alignment, and tissue stiffness in the context of obesity and breast cancer.

Our evaluations of 3-D and 2-D collagen fiber morphology suggest that metrics that are sensitive to the 3-D orientation of the fibers are more sensitive than ones that exploit only 2-D image features. This finding is consistent with previous studies in which we performed more direct comparisons of the orientation variance of the fibers in 2-D and 3-D contexts and showed that the 3-D-based analysis was a more sensitive reporter of subtle changes.^16^^,^^18^ Nevertheless, 2-D images are typically more readily available than 3-D image stacks, and it is both important and useful to evaluate complementary methods of analysis.

For our studies, we examined breast tissues that had been flash frozen immediately upon excision from mice. Obesity was induced in mice through the introduction of an HFD, and tumors were induced through the implantation of EO771 cells, which were derived from a spontaneous adenocarcinoma from a C57Bl/6 mouse. These tumors grow faster than typical transgenic models of breast cancer and are not typically associated with a significant stromal component, which is consistent with the overall decrease in fiber density that we observe within the tumor tissues. However, it will be important to extend our studies to different breast cancer models to determine whether we observe similar trends in terms of the impacts of obesity. Also, some degradation, especially in the CAF signal, and structural changes are possible upon freezing and thawing of the tissue. Since, we followed identical tissue handling protocols for all samples, we anticipate that, even if present, such processing-induced changes would impact in a similar way all our samples and would not affect our main results that focus on matrix differences between groups. In addition, previous studies performed with frozen-thawed chick embryo tendon tissues, indicated a correlation of CAF intensity with the levels of LOX-mediated crosslinks assessed by mass spectrometry.^20^ Measurements that are performed *in vivo* either through probes or minimally invasive procedures that may expose the tissue would overcome these limitations. Finally, as with any high-resolution imaging technology that samples a limited region of the tissue, there are always concerns that the regions selected for sampling may result in biased assessments. To avoid introducing any potential bias in our calculations, we made an effort to sample the diversity of features present in every sample visualized following a 2-D tile scan of a large (5×5  mm) tissue area.

In summary, our study demonstrates the potential of label-free two-photon imaging to assess complementary features of collagen fiber organization and structure and to provide useful and highly quantitative insights regarding the remodeling processes that occur in breast cancer. Exploitation of the 3-D nature of the images we acquire using SHG imaging enhances our sensitivity to potentially subtle changes that may remain undetected in some cases if the analysis is limited to 2-D images. Acquisition of SHG and TPEF images simultaneously is possible and can provide information regarding multiple aspects of collagen organization and structure, all of which may ultimately play an important role in affecting cell behavior. Thus, their combination could be a powerful tool in helping us to understand better the complex role of risk factors, such as obesity, in the development and progression of breast cancer.
