# Supplementary material for: Factors associated with obesity alter matrix remodeling in breast cancer tissues
Source: J Biomed Opt. 2020 Jan 25;25(1):014513. doi: 10.1117/1.JBO.25.1.014513 (PMC6982464; doi:10.1117/1.JBO.25.1.014513)
Supplement: Supplementary file 1 [file JBO_025_014513_SD001.pdf]

SUPPLEMENTAL INFORMATION FOR

Factors associated with obesity alter matrix remodeling in breast cancer tissues

By Yang Zhang,[a] Fatma Kucuk Baloglu,[a,b] Lauren E. Hillers Ziemer,[c] Zhiyi Liu,[a,d] Boyang Lyu,[e] Lisa M. Arendt,[c] and Irene Georgakoudi[a,f,*]

[a]Tufts University, Department of Biomedical Engineering, Medford, Massachusetts, United States

[b]Department of Biology, Giresun University, Giresun 28200, Turkey

[c]University of Wisconsin-Madison, Department of Comparative Biosciences, Madison, Wisconsin, United States

[d]Zhejiang University, State Key Laboratory of Modern Optical Instrumentation, College of Optical Science and Engineering, Hangzhou, Zhejiang, China

[e]Tufts University, Department of Electrical Engineering, Medford, Massachusetts, United States

[f]Tufts University, Program in Cell, Molecular & Developmental Biology, Graduate School of Biomedical Sciences, Boston, Massachusetts, United States

Supplementary Note #1: Alternative view of Fig. 1c is shown in Fig. S1, along with an expanded view of the dotted region from a different orientation to visualize more clearly the transverse orientation of the collagen fibers within that region.

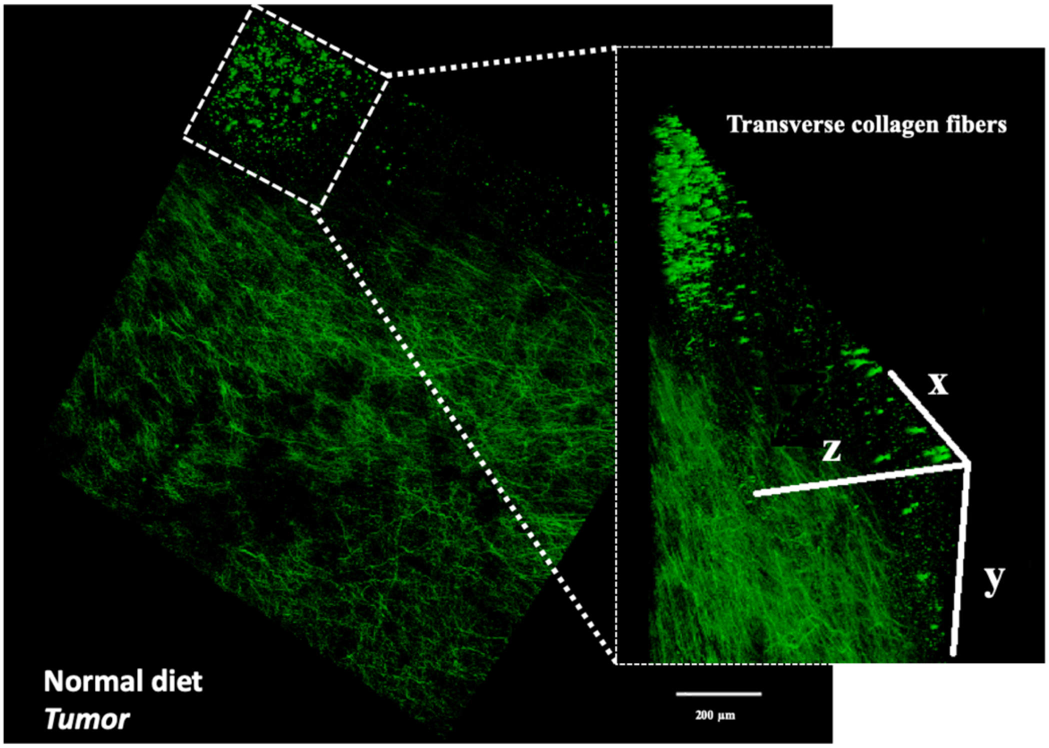

Figure S1. Transverse collagen fibers in the 3D volume rendering of NT group (Normal diet Tumor).

Supplementary Note #2: Fiber thickness and density are important factors that can affect the accuracy of the average fiber orientation measurement and the size of the optical window over which orientation and 3-D directional variance is calculated can affect the sensitivity with which differences can be assessed. For the results reported in Figure 2, we chose a 5.3x5.3x6 μm^3 voxel, which results in significant differences not only between tumor and normal tissues, but also between tumors from mice fed with either a normal or a high fat diet, as indicated in Fig. S2. The ability to detect significant differences among some of the group decreases when the full stack (387.5x387.5x 80 μm^3) is used for 3D directional variance calculations.

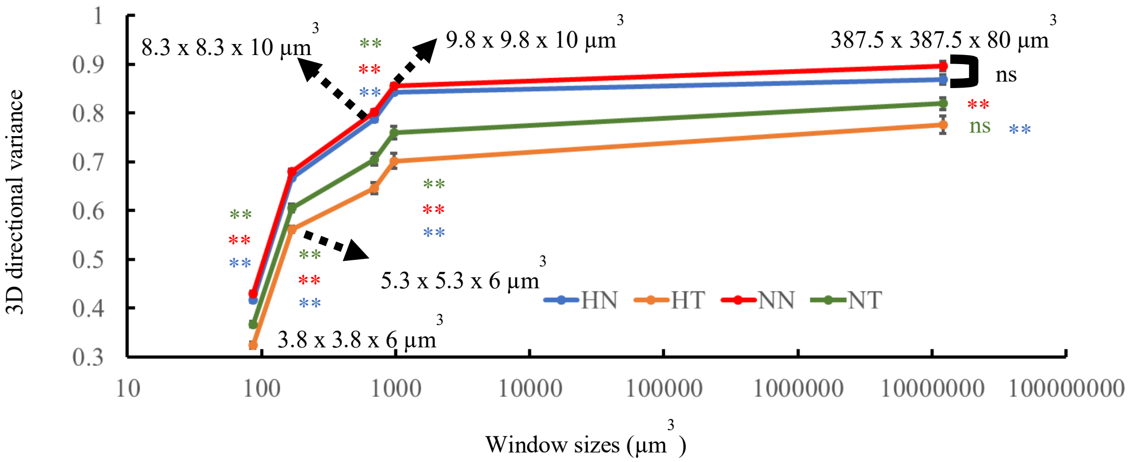

Figure S2. Mean and standard deviation of 3D directional variance as a function of window size. Significant differences between healthy and tumor groups are detected for window sizes equal to or larger than 3.8 x 3.8 x 6 μm^3. The last assessment window 387.5 x 387.5 x 80 μm^3, corresponds to the entire 3D image. ** denotes a= 0.01.

Supplementary Note #3: The 3-D directional variance PDFs can vary significantly among samples even of the same group as shown in Fig. S3. In the tissues from non-tumor bearing mice some consistent differences are noted in the PDFs between fiber rich and duct/fatty regions for both the normal and high fat diet groups (Fig. S3 A-D). However, for the tumor tissues, we observe wide variations in both diet groups that don't appear to be associated with the level of cellularity or collagen fiber density (Fig. S3 E-F).

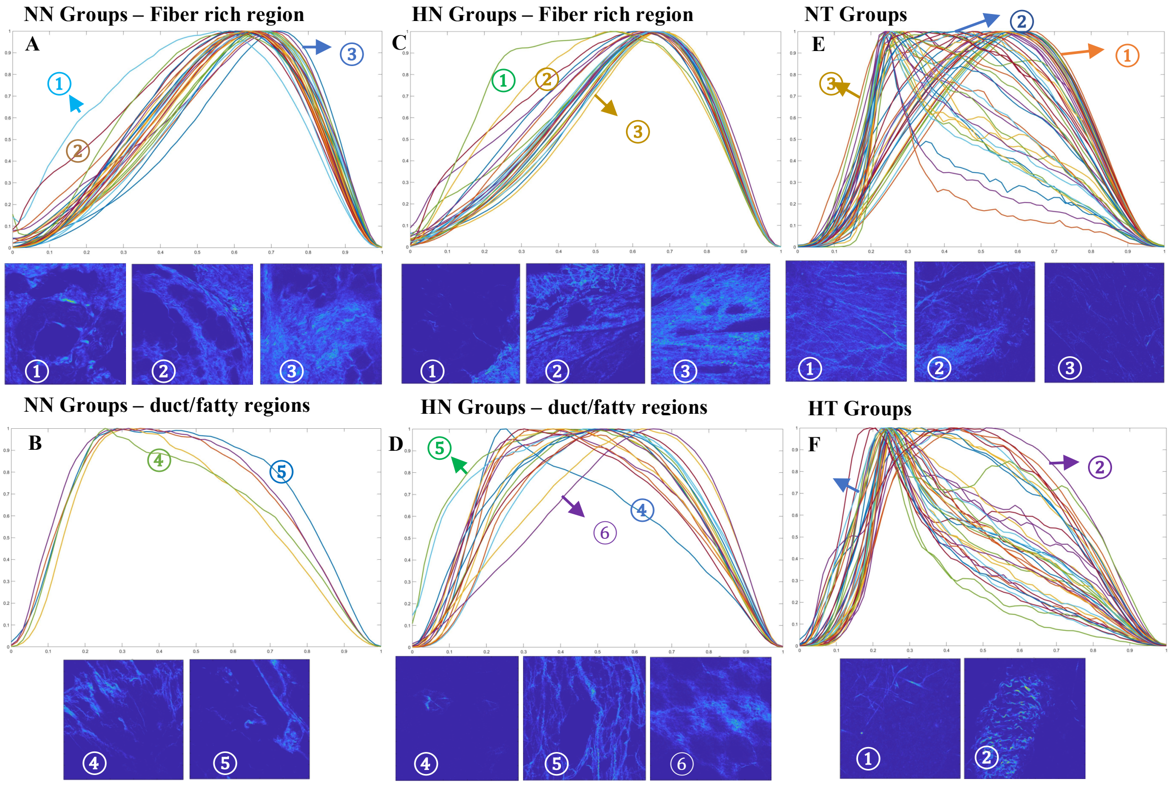

Figure S3. Different tissue morphology alters the variance distribution shape. Each panel shows the PDFs of the 3D variances of each field and representative tissue features per group. Distributions with obviously different shapes are numbered and the corresponding tissue features are listed below each panel for comparison. A. PDFs of stacks that have high fiber content for NN group (Fiber rich region). B. PDFs of stacks that mainly contain adipocytes or mammary ducts in NN group, and have significantly different alignment characteristics relative to the fields in A; C. Fiber-rich regions of HN group; if the image stacks contain large quantities of adipocytes, the distribution is typically broader (same as A1) D. PDFs of stacks that mainly contain adipocytes or mammary ducts within HN group; E. All PDFs for NT group; Distributions are left-shifted compared to non-tumor groups. F. All PDFs of HT group; the distributions are even more left-shifted compared to the distributions in E.

Supplementary Note #4: Even though mice fed with the high fat diet gained significantly more weight and had tumors that were larger, as indicated in Fig. S4 A, B, the tumors of the ND and HFD groups appeared similar following H&E staining (Fig. S4 C, D) or α-SMA staining for cancer associated fibroblasts (Fig. S4 E, F).

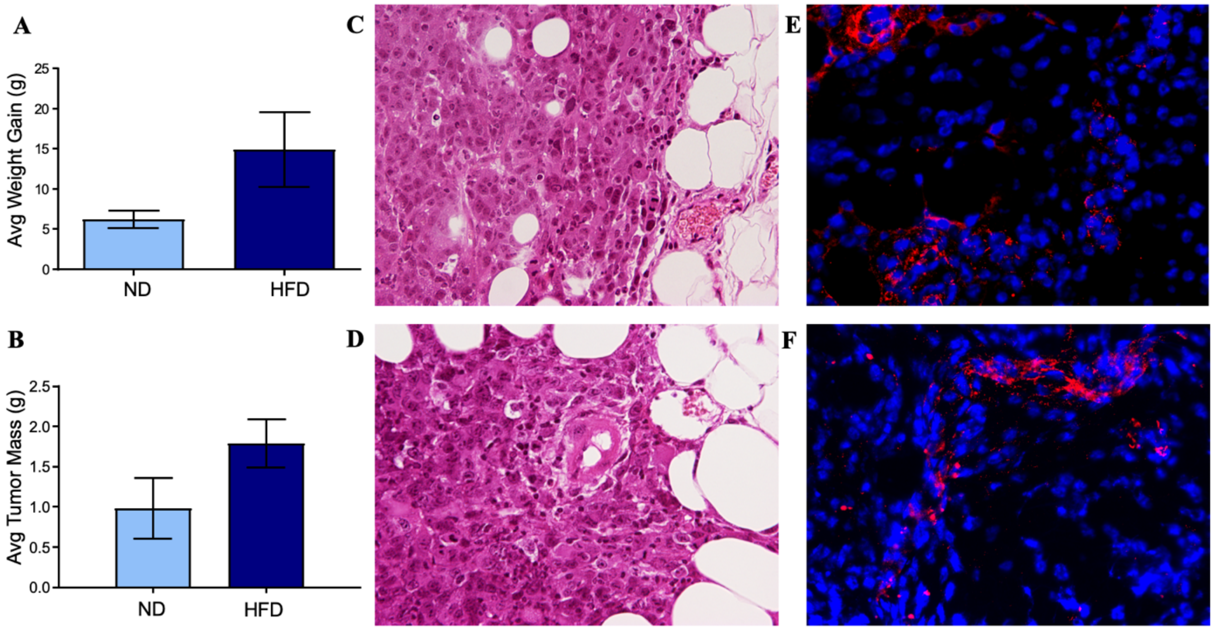

Figure S4. (A) Average weight tumor and (B) tumor mass from tumor bearing mice fed either a normal (ND) or high fat (HFD) diet. Representative H&E (C,D) and aSMA (E,F) -stained sections from tumors from (C, E) ND and (D, F) HFD mice.

Supplementary Note #5: The differences in collagen fiber density reported in Fig. 4 are presented in Table 1.

Table 1. The maximum, upper quantile, median, lower quantile, and Minimum of the fiber density data of each group. Box plots can be seen in Fig. 4.

**Fiber density**	**NN**	**HN**	**NT**	**HT**
**Maximum**	0.8761	0.5914	0.5102	0.1315
**Q3**	0.3401	0.4289	0.1811	0.0141
**Median**	0.1952	0.2928	0.0194	0.0068
**Q1**	0.103	0.0494	0.0045	0.0036
**Minimum**	0.0228	0.0144	0.0005	0.0001
